# Social inclusion and violence prevention in psychiatric inpatient care. A qualitative interview study with service users, staff members and ward managers

**DOI:** 10.1186/s12913-021-07178-6

**Published:** 2021-11-20

**Authors:** Veikko Pelto-Piri, Lars Kjellin

**Affiliations:** Faculty of Medicine and Health, University Health Care Research Center, Örebro University, 70182 Örebro, SE Sweden

**Keywords:** Social inclusion, Values, Policy, Psychiatry, Inpatient care, Violence prevention

## Abstract

**Background:**

Many psychiatric services include social inclusion as a policy with the aim to offer users the opportunity to participate in care and to form reciprocal relationships. The aim of this study was to explore opportunities and problems with regard to participation, reciprocity and social justice that different stakeholders experience when it comes to social inclusion for service users and minimizing violence in psychiatric inpatient care.

**Methods:**

Qualitative interviews were performed with 12 service users, 15 staff members, and six ward managers in three different kinds of psychiatric wards in Sweden. The data were analyzed using the framework method and qualitative content analysis, which was based on the three following social inclusion values: participation, reciprocity, and social justice.

**Results:**

Themes and subthemes were inductively constructed within the three social inclusion values. For participation, staff and ward managers reported difficulties in involving service users in their care, while service users did not feel that they participated and worried about what would happen after discharge. Staff gave more positive descriptions of their relationships with service users and the possibility for reciprocity. Service users described a lack of social justice, such as disruptive care, a lack of support from services, not having access to care, or negative experiences of coercive measures. Despite this, service users often saw the ward as being safer than outside the hospital. Staff and managers reported worries about staffing, staff competence, minimizing coercion and violence, and a lack of support from the management.

**Conclusions:**

By applying the tentative model on empirical data we identified factors that can support or disrupt the process to create a safe ward where service users can feel socially included. Our results indicate that that staff and service users may have different views on the reciprocity of their relationships, and that users may experience a lack of social justice. The users may, due to harsh living conditions, be more concerned about the risk of violence in the community than as inpatients. Staff and ward managers need support from the management to foster a sense of community in the ward and to implement evidence-based prevention programs.

**Supplementary Information:**

The online version contains supplementary material available at 10.1186/s12913-021-07178-6.

## Background

The United Nations’ 2030 Agenda for Sustainable Development has social inclusion at its core, and it is an important policy in the European Union [1–4]. The theory of social inclusion is broad and covers economic, social, and political dimensions. Social inclusion can be described as processes that work towards social cohesion, which in turn have positive effects on the health of a population [5]. To work according to social inclusion can be defined as a process in which opportunities are offered, especially to people who are disadvantaged, to participate in a community, offering access to resources and a voice in society. On a community level, social inclusion allows people to participate in activities, thereby enabling them to form reciprocal relationships with others. Social inclusion is not only a theory, it is often used as an explicit policy and goal for psychiatric rehabilitation in social and mental health services. It is however not yet clear what constitutes social inclusion or social exclusion, thus making it difficult to measure [5–8]. There are different discourses in research of social inclusion theory, like the social integrationist discourse seeing inclusion mainly as a paid employment [9]. This perspective has been criticized by other researchers, that social inclusion can be defined normatively, as the achievement of a mainstream lifestyle and ideals [5]. This may lead to moralistic judgments and oppression against people who choose an alternative lifestyle or who reject offers made by services [2, 5]. Therefore, the concept of social inclusion is not completely clear, and we should accept that it can differ depending on the context and individual [5]. While it might seem that social inclusion and social exclusion are one phenomenon, a continuum from exclusion to inclusion, it is more fruitful to see them as overlapping concepts. Social exclusion is synonymous with inequality and the aim is to identify and eliminate barriers and stigmatic situations for disadvantaged persons [9]. But only by eliminating these problems does not guarantee that people will feel included. The aim of social inclusion is to find ways to increase opportunities for service users to be included whether there are barriers and stigma or not [3]. Psychiatric and social services have often been criticized for socially excluding service users; for example, psychiatric inpatient care in Europe has been criticized for over-using coercion and detainment [10].

Faced with this it is relevant to ask in what way the restrictive environment of a psychiatric ward can be socially inclusive. The staff must balance the requirements for a positive risk-taking rehabilitative approach with safety measures to prevent hazardous incidents [11, 12]. Prevention programs, such as Safewards and Six Core Strategies, have given recommendations that are, in their essence, very similar to a human rights-based approach to psychiatry, the social inclusion theory, and a recovery approach when considering the service user’s viewpoint and communication between stakeholders [13–16].

Clifton et al. [7] included *participation, tackling discrimination,* and *giving opportunities* as dimensions of social inclusion. Health care in Western societies has a policy of enforcing patients’ rights to *participation* in their care and treatment, and the World Health Organization (WHO) recommends a participatory approach in violence prevention [17]. Two important features of participation are 1) the service users can be involved in their care and other issues affecting them, and 2) the staff–service user interaction is characterized by empathy and respect, even in situations that are emotionally challenging for staff [12, 18, 19]. Service user participation is a well-known concept and can refer to relatively passive involvement by the service user [20]. Nonetheless, research has indicated that adopting participation as a policy has little influence on clinical practice; for instance, service providers do not always trust service users’ capacity to make decisions, especially if their suggestions are not in line with the providers’ views [19].

We think that *Tackling discrimination* conceptually belongs to the theory of social exclusion, to work against inequality. Clifton et al. [7] describes *Tackling discrimination* as a process of promoting control and hope by facilitating control over own symptoms and problems in order to develop and practice new skills in new situations. To us, this seems to be a work style aiming at recovery and working close to the person based on *reciprocity,* a concept closer to social inclusion theory than tackling discrimination. If the service users are participating, their relationship with staff can develop into reciprocity. In this case, the service user is seen as a person and not merely as a patient. In a reciprocal relationship, it is not only the competence of staff that counts, but also that of the service user [21]. Shared decision making and person-centered care align with these values [22]. Indeed, these two methods make it possible to work from a recovery perspective, go beyond psychiatric symptom management, and together design strategies for the service user to cope with symptoms and problems encountered in life [7]. One way to support recovery-oriented work is to ensure that the ward develops a good social community [13]. This kind of development in psychiatry is recommended in the guidelines of the National Institute for Health and Care Excellence because it may contribute to the reduction of violence [12]. Overcoming the power imbalances can be a challenge for staff members when they want a have a more reciprocal relationship with service users and their network [23, 24]. If staff are unable to maintain a relationship with the user, they might rely on coercive measures rather than alternative interventions such as calming and de-escalation techniques [25, 26].

The third dimension, *Giving opportunities,* is described by Clifton et al. [7] as a process promoting rights and equality. In our opinion, providing opportunities can be seen as part of a broader value of “social justice”, which implies that service users are right-holders as citizens and can make legitimate claims against psychiatric and social services, who are the duty-bearers [15]. *Social justice* addresses the political dimension of care, wherein the service user has the right to good psychiatric services in which they are not exposed to discrimination, violence, coercion, or detainment [9, 27]. The use of coercion could be reduced through staff training and a program for restrictive intervention reduction, which aim to transform the ward into a positive community with access to meaningful activities [12, 25, 28, 29]. The method of co-creation can be used to encourage all stakeholders to work together in designing and implementing safe, inclusive, and user-friendly services [17, 20, 30]. Co-creation between staff members and service users has been used to implement the violence prevention program Safewards [31]. One of the ten Safewards interventions is called mutual expectations, which replaces ward rules that only apply to service users [25, 32]. In this intervention, staff and service users design these mutual expectations together.

Based on the literature presented above and in dialogue with the Social inclusion framework of Clifton et al. [7] we thus have identified three essential values in social inclusion: participation, reciprocity, and social justice and created a tentative model of social inclusion for psychiatric inpatient care. (Table 1).

**Table 1:** A tentative model of values in social inclusion and violence prevention: participation, reciprocity, and social justice

Values	Service user seen as a	Perspective	Goal
Participation	Patient	Patient’s rights	Informed patients influencing the care given by staff.
Reciprocity	Person	Dialogical	The knowledge of both service user and staff is used to create recovery strategies.
Social justice	Citizen	Human rights	Access to user-friendly and non-discriminating services.

Since social inclusion is not only a theory but used as a policy it can affect how psychiatric services are delivered, and thus it is important to know how different stakeholders reason about opportunities and problems around social inclusion. There is also a theoretical interest in understanding the mechanisms that can support more socially inclusive mental health services [9]. Clifton et al. [7] did not test their model of social inclusion empirically. While social inclusion can be integrated with the prevention of violence, no empirical research has focused on social inclusion in psychiatric inpatient care. In the present study we intended to analyse data from an ongoing study on violence in psychiatric inpatient care, looking for expressions of the three values in our tentative model. Thus the aim of this study was to explore opportunities and problems with regard to participation, reciprocity and social justice that different stakeholders experience when it comes to social inclusion for service users and minimizing violence in psychiatric inpatient care.

## Methods

### Design

In our project Prevention of violence in psychiatric inpatient care – aspects of ethics and safety, we have interviewed service users, staff members and ward managers in psychiatric inpatient care. We have published two descriptive articles using most of this data with a qualitative content analysis with an inductive approach focusing on stakholders own perspectives [33, 34]. These articles give a description of how 1) staff and managers perceived aspects of safety and ethics [33] and 2) patients perceived safety and unsafety at the wards [34]. In the present study, using a deductive content analysis, the focus is on opportunities and problems around social inclusion reported in the interviews. Deductive content analysis is useful in when retesting existing data in relation to a model [35]. In the present study, we performed a deductive analysis with an analysis matrix containing participation, reciprocity, and social justice [35]. Thereafter inductive approach was used in order to create themes and subthemes [35]. The framework method was used to organize the process and data in order to get a summary of the results in a framework [36].

### Settings and participants

The setting was three wards of inpatient clinics for adults in three different regions of Sweden; one was a general psychiatric clinic, one was a psychiatric addiction clinic, and one was a medium security forensic psychiatric clinic. The general psychiatric and psychiatric addiction clinics cared for patients in need of psychiatric inpatient care from the respective surrounding catchment areas, and the forensic clinic also had patients from other areas of Sweden. Substance users could be found in all three clinics, but the psychiatric addiction clinic was specialized to care for patients with a combination of addiction and psychiatric diagnoses. The purpose of this selection was to obtain a wide range of clinics. At the time of the study, all wards intended to provide single rooms for all patients.

We interviewed 12 service users, four women and eight men who were aged from 23 to 67 years (median 47 and mean 46,7 years). The inclusion criteria were that the service users should not be too ill for an interview, could speak Swedish, and had been cared for in inpatient care for long enough to have experiences to convey; indeed, most had a history of several admissions. We also recruited service users to obtain a mix of both sex and age.

The study comprised three focus groups with three nurses and twelve assistant nurses. Four to six staff members participated in each focus group, with a total of 15 participants, ten women and five men. Nine of the staff members had at least 10 years of experience of institutional care, the four staff members with least experience of psychiatric inpatient care had worked 1,5–4 years. We have no information about the actual training of the participants, but the forensic psychiatric ward had a 2-day course in de-escalation and physical techniques with regular repetitions. The general psychiatric ward had a 2-day course, too, but with less systematic use, while the addiction clinic did not have a decided training program for staff. While data were being collected, a change of manager occurred in all three wards; as such, we interviewed both the outgoing and new manager, resulting in a total of six ward managers, three women and three men and half of these had more than 10 years of experience. One of the managers was new to psychiatric inpatient care, he chose to do the interview together with the outgoing manager who had long experience.

### Procedure and interview guides

Patients and staff were recruited by the ward managers, who gave them verbal and written information about the study. In the semi-structured interview guide for service users, the main question was about the service users’ perceptions of feeling safe or unsafe in the ward. There were also some optional questions about how the service user perceived the importance of (1) the ward’s physical design, (2) the ward’s routines and rules, (3) the staff’s approach to service users, and (4) the presence of other service users in relation to feeling safe or unsafe. We also asked about situations they perceived as threatening or violent. Interviews lasted around 50 min. We did some test interviews at Fountain house in Örebro when constructing the interview guide for service user, for interview guide, see Pelto-Piri et al. [37].

Staff and ward managers completed a semi-structured interview that included the four following central question areas: (1) values at work, especially regarding interactions with the service user in general, (2) safety questions in general, (3) the concrete management of situations that were violent or expected to present a risk of violence. The interviewer had the task of encouraging people to give their views on aspects of values and safety in violence prevention and ask follow-up questions about organizational support. The interviews with the managers were adapted to a managerial perspective. Each interview lasted approximately 60–90 min and was conducted by one interviewer and one observer from the research team. For interview guides, see Additional file 1.

The clinics we wanted to include in the study chose to participate and all ward managers participated after written and verbal information. All recruitment of other participants in the study was done by ward managers, which means that we do not know how many people declined to participate. The majority of the interviews were performed in 2014–15 by the first author and five other members of the research team: a psychiatrist, two psychologists, an educationalist and an investigator. The participants did not have a previous knowledge of the researchers or their ambitions or reasons for doing the research. We informed that this was research on violence where we were interested in aspects of ethics and safety/security. All interviews were single sessions and audiotaped, they were conducted in the psychiatric wards and only the participants and researcher(s) were present, field notes were not used. All interviews were audio recorded and transcribed verbatim, the transcripts were not returned to the participants for comments. Preliminary findings were discussed at two seminars. One with previous patients from the same admission area and another with managers and staff from the clinics who were included in the study.

### Analysis and interpretation

We used the framework method according to Gale et al. [36]. For the analysis procedure, seven recommended stages were followed.

1. *Transcription.* A verbatim transcription of the recording was made by a professional transcriber.
2. *Familiarization with the interview.* We read through the interviews while focusing on social inclusion and violence prevention.
3. *Coding.* We chose to start with three interviews from general psychiatry, the focus group interview with staff, and a manager and a user interview which, according to our initial assessment, had a rich content in relation to our aim. Statements relevant to social inclusion and violence prevention (Table 1) were marked as a meaning unit, and each unit was placed in one of the values in the analysis matrix, they were assigned a code and/or a brief description that was as close to the content as possible [35]. Excel was used to organize codes and themes.
4. *Developing a working analytical framework.* Themes and subthemes were created inductively from deductively chosen meaning units, whereby meaning units were organized under higher-order headings [35]. After coding the first three transcripts, we compared the labels to which they applied and agreed on a set of preliminary themes. These results were presented and discussed at a workshop with a psychiatric network that included humanities and social sciences researchers. After this meeting, a preliminary analytical framework was formed according to social inclusion theory and from our preliminary results.
5. *Applying the analytical framework.* A framework with the three values, participation, reciprocity and social justice, and five themes within these was applied to all interviews.
6. *Charting data into the framework matrix and reducing data.* The first framework matrixes included one for each of the three settings, as follows: a) each matrix had one line per interview with three columns for the values, b) each matrix was reduced to three lines, consisting of service users, the focus group, and the ward managers, and c) all three matrixes were merged into one matrix for all settings, with one line for each stakeholder group and three columns, one per value.
7. *Interpreting the material.* The interpretation work began by checking the selected themes and investigating what other themes needed to be created within the values. At this stage, we started to work in English. A preliminary result was presented at a conference. After the conference, we continued reading the literature and interpreting the material. The process was finalized by critically examining the content of the selected themes/subthemes and their names.

All material was available throughout the analysis, so we could go between the whole interviews and our categorization of the text. Both authors participated in all phases of the analysis. Since this is a qualitative interpretive analysis it is not applicable to measure inter-rater reliability [38]. We have thoroughly discussed different interpretations until we have reached consensus [39].

## Results

During the analysis of the empirical material based on the social inclusion values of participation, reciprocity, and social justice, we constructed the five following themes: 1) patient involvement in care and treatment, 2) sense of community, 3) us and them, 4) access to good care, and 5) quality of care. Within these themes, 13 subthemes were constructed (see Table 2).

**Table 2:** Themes and subthemes within the model of three social inclusion values; participation, reciprocity, and social justice

Values	Themes	Subthemes
Participation	Patient involvement in care and treatment	Difficulties in engaging patients in care and treatment. Sensible communication between patients and staff.
Reciprocity	Sense of community	The ward as a meeting place. The patient community as a resource.
	Us and them	The health care hierarchy. The tough jargon. Negative views of the psychiatric patient. The large differences in living conditions.
Social justice	Access to good care	To get access to care. To be allowed to stay in care.
	Quality of care	Service users seeing the ward as an asylum. Managers and staff concerns about adequate staffing and competence. Minimizing coercion, violence, and injuries.

### Participation

#### Patient involvement in care and treatment

##### Difficulties in engaging patients in care and treatment

Staff and ward managers described patients who required extensive care and treatment; in particular, substance users were vulnerable and suffered from, for example, starvation symptoms or diabetes. Staff and ward managers considered it important, but difficult, to motivate service users and get them involved in their own care, especially service users who were seriously or chronically ill. In a discussion about service user involvement, one staff member recounted the following:

Yes, it’s really varied. We have a patient that has never been in psychiatry. He really wants to get out, so it’s very easy to engage him. Then we have someone who has been here for 14 years and he is probably not as easy to motivate. (Staff 3)

Service users were often not confident in the content of their care. They could not always understand the care given, such as why they were not being given the same medication that had worked well during their previous care episode, or why withdrawal of a medication had to be done so quickly that it resulted in serious side effects. Service users were more often worried about the long-term care and support than the treatment received at the ward. A service user with many disappointments from previous psychiatric inpatient treatment had a more positive experience during this episode of care.

Obviously, I don't want to take up a place for someone who is sicker than I am. But, to avoid coming back, I’ve got to form a care plan together with the social services - and I’ve now been promised that this will happen before I go home. (Service user 1)

##### Sensible communication between patients and staff

Ward managers and staff expressed a wish for staff to be more open to service user participation and to ask service users how they wanted to interact with staff. One ward manager emphasized that staff members need to participate more in the care of service users to get service users involved, but that not all staff were interested in doing so. According to managers and staff, there were differences in staff members’ care styles of interaction with service users: some used “rigid old-fashioned frameworks”, others a more modern “flexible way”.

You don’t have to go in [to the patient] and be adamant that this is what we’re going to do. Instead, ask the patient: “How do you think you could handle your anxiety?” and “What do you think you need right now”. I think that there has been some change here lately, but, at the same time, we have these rigid old frameworks; if you scratch the surface a little, you’ll see a “we decide” attitude. I think that’s wrong. (Manager 2)

Staff members reported being intrigued by service users’ stories (“It’s like a different world, so you listen when they speak”). Many service users seemed to appreciate talking to them, and staff could recognize the ability and willingness of patients to do so.

This is something that I’m impressed with in our psychiatric patients, that they are so good at talking; talking about how they are and how they feel. Haven't you reflected on that? (Staff 3)

Staff also reported apologizing to service users when they noticed that they had unnecessarily violated the service users’ personal space or privacy.

Service users who experienced anxiety noticed and appreciated when staff members talked with them in a calming tone instead of using coercion, but they also noticed that some staff members showed disinterest or communicated negatively with service users. Service users were expected to behave calmly when communicating with staff members.

That’s it! For goodness sake, you must be allowed to show your feelings. I mean, anger, it’s a feeling; you get angry about something. Yes. I mean, that’s normal […] It doesn’t work in places like this. Then you get put in strap restraints, for heaven’s sake! (Service user 10)

### Reciprocity

#### Sense of community

##### The ward as a meeting place

Ward managers wanted to create a good social atmosphere, and to keep the ward safe and secure. They worked with team ethics, not only on how team members behaved towards each other, but also in their interactions with service users. One ward manager thought that staff needed to be more visible in the ward. However, inspiring change in staff behavior is a delicate mission. Staff and ward managers highlighted the relations with service users as important. Long and good relationships were reported to contribute to workplace safety, and enabled staff members to be much more direct and tough in critical situations.

After all, I have patients that I have known for 10 years who I can go in to and say, “That’s enough, you!” in a sharper tone. “I don’t accept your behavior” (Manager 1)

Forming staff–service user relationships enables staff to more easily recognize if service users are not capable of handling their emotions. Sometimes, service users protected certain staff members from themselves by warning them of feelings they might not be able to control.

Some service users described a sense of community, whereby both staff members and service users openly socialized and communicated.

Other times, you sit in the TV room or on the sofa, talking and joking, exchanging experiences; patients and staff alike. Teasing the staff a little. Ha ha ha – teasing or joking with them. They’re just the same themselves too, so I would say it’s perfectly normal. (Service user 5)

Many other service users did not perceive this familiar atmosphere, and service users frequently had to take the initiative to communicate with staff. Sometimes, service users felt that they should not make contact since it might interfere with the staff’s work.

##### The patient community as a resource

According to service users, the patient community could be an important resource in recovery, whereby service users who had experienced progress in their rehabilitation could support other users. Sometimes, a service user would apologize to another service user if they had behaved in a frightening way. They saw it as their duty to talk to these silent co-users when no one else, staff or other users, was talking to them.

And then, of course, I’m a pretty verbal person. But there might be those who are not so verbal; just like Fredrik out there. He doesn’t say so much at all, so there might be even less communication than with others. I haven’t seen anyone talking to him. I notice that he comes and sits down where I sit and I talk to him. I ask him “How’s it going, Fredrik?”, and he says, “It's good” and tells me where he works. It’s maybe not that easy, so the staff need to take the first step, but I haven’t noticed them do that. (Service user 2)

The service user community could also be seen as problematic; old conflicts with other service users and very ill, anxious, or intrusive service users could result in anxiety and absconding. Service users very seldom received information on how staff would handle probable future incidents, which made them uncertain about the security of the ward. A female service user avoided some male co-users because she considered them to be “women haters”. When the staff brought up the topic of the service user community, it was about problems with managing the service users, not how the patient community could be used as a resource.

#### Us and them

##### The health care hierarchy

Staff who had worked in psychiatric wards for decades described the psychiatric wards as very hierarchical, not only for service users, but also for staff. All stakeholders thought that psychiatry is now developing in the right direction; less towards hierarchy and more towards humane attitudes. However, service users were well aware of the organizational hierarchy and that care staff could not always meet service users’ needs, since they did not have the power to decide.

No, there’s one thing I hope we don’t forget – that those who work on the floor here at the ward get their directives from above, and we don’t meet these people. They have to follow these directives, so their hands are tied – and I can fully understand it. (Service user 8)

##### The tough jargon

Ward managers, but also staff, were aware of the jargon between staff, and between staff and service users, as well as staff members’ high tolerance of verbal threats. It was important for the ward managers that staff and service users should not use language that is not tolerated in society. It is considered as part of the rehabilitation of service users, especially in forensic psychiatry.

It’s actually at that level that someone can go by and say, “I’ll shove your head through the wall, you bastard”. “Yeah, yeah, let’s sit down and watch TV”. It’s at that level. It is a very high level of tolerance that I don’t find acceptable at all […] it’s not acceptable that you basically get four death threats within the first quarter of an hour because they had a bad morning. The level of tolerance is insanely high. (Manager 5 & 6)

##### Negative views of the psychiatric patient

Service users reported feeling less worthy than other people when they were in a very bad condition, not having the strength to live any longer, and experiencing feelings of guilt and shame.

Yes, what happens is you feel less worthy because you’re, like, because you’re sick or because it’s an illness. So yes, it didn’t feel like anything was serious. It just became like a big, just a big playground because no one, no one heard. (Service user 9)

These feelings became stronger if a staff member indicated that their situation was self-inflicted, which substance service users were especially sensitive to. A service user chooses to let the police pick him up when he needs to get to the hospital since the ambulance might have more important patients than “a psycho patient”.

One ward manager complained that some staff members and physicians did not like to work with substance users. Stakeholders noticed that staff members, consciously or unconsciously, sometimes showed that they did not like this type of service user.

##### The large differences in living conditions

Many service users had harsh living conditions and perceived there to be a greater distance between themselves and the staff when differences in living conditions between them became apparent. Service users did not want to listen to staff members talk about aspects of their private life that were inaccessible to them, such as trips to Thailand or Spain. Some service users had been homeless for long periods of time, and one woman had asked the social services to take her child to a new home, since she was aware that she did not have the resources or capability to take care of her child.

He shouldn’t go back and forth and social services shouldn’t piss on me and harm my child. It should be a family home that already has children and I should have custody and visitation rights. But he shouldn’t live with me. He should have a good life… he should have a chance at life. And that’s what happened. That’s how I worked it out. (Service user 11)

### Social justice

#### Access to good care

##### To get access to care

Service users were disappointed with the possibilities to get access to health care and described many incidents of delayed care.

This is what I feel, a disappointment towards society and health care, that I haven’t got the right help from the beginning – that I had to come in, be convicted of a crime – that you have to go as far as being convicted of a crime before getting any help. (Service user 12)

The service user cited above stated that he did not get adequate treatment until he had been convicted to forensic psychiatry, and was disappointed with society, also on behalf of the victim of his crime. Service users wanted staff in the emergency room to recognize their needs and admit them to the ward.

[…] when she says, “No! You’ve got to go!”. That's not what I want to hear. I want to hear something like, “Stay here”, and be given these words instead. Because I don’t want to leave. I’ve had tears in my eyes […] just getting that word “Go!”. I’ve heard that all my life. (Service user 8)

This service user spoke about how he could be verbally aggressive in these situations and apologize afterwards for his behavior. Access to care was a problem for many service users, an issue rarely raised by the staff and ward managers. Only one manager was worried about recurrent patients, as these patients are probably not receiving adequate care and support, yet at the same time, these patients used a large share of the treatment resources.

##### To be allowed to stay in care

A problem for ward managers was that certain service users were not welcome on their ward, and the staff argued that these service users should be cared for by another ward or caregiver.

When fear sets in, there’s a lot of uncertainty among the staff group and they want to try to push this patient away - This is actually PICU’s [Psychiatric Intensive Care Unit] patient. PICU has to take this now. But PICU is full and we should be able to deal with things like this too. No, this is actually PICU’s case! What do we have PICU for? - So they want to get rid of the problem, I think. (Manager 3)

Ward managers reported that there was sometimes a risk of service users being discharged despite a continuing need for care if the staff were afraid of violence from the service user.

Many service users were worried about what would happen after discharge. They did not want to make demands or “be awkward” towards staff, despite their awareness of an acute need for care or treatment; in these cases, service users were afraid of being too demanding because they thought it would result in punishment or not being admitted into care next time they might need it. Service users sometimes felt that they were “discharged to nothing”. They often wished for a concrete care plan that also considered social aspects, including housing and work, and few service users had a care plan that they believed would work.

#### Quality of care

##### Service users viewing the ward as an asylum

An important quality of care that many service users emphasized was viewing the ward as an asylum that offered shelter from violence in society and the weather, especially in winter time, and as a place where there was plenty of food.

Yes. There’s breakfast, lunch, coffee, dinner, and even evening coffee. So there are two cooked meals a day. And then the worst thing is striking a balance, getting used to it when I leave, because I don't eat that much when I’m at home. Ha ha! (Service user 5)

One service user expressed that, as a service user, you have to be grateful for the opportunity to be admitted to the ward. Service users were grateful for the staff who cared for and monitored them, and reported that it felt safe to have nurses and physicians present.

##### Managers and staff views about adequate staffing and competence

In the interviews, the staff and managers focused on the quality of care in terms of the competence of staff in relation to diagnostic groups and the management of coercive measures and violence. Managers emphasized the importance of a stable and experienced nursing team.

The staff is the most important resource we have in our work. It is the work tool we have to work with. We can certainly give medicines and we can do other things, but if we don’t have human resources who treat people properly and give a good response and create peace and quiet and security, then there are no drugs in the world that can replace it. (Manager 4)

Both staff and ward managers reported wishing that they had more support from the higher management with staffing and competence levels to be able to give a better quality of care. A lack of experienced staff can result in more serious injuries and the use of coercive measures in an earlier stage of conflict.

##### Minimizing coercion, violence, and injuries

Ward managers and staff reported improvements in their competence at handling difficult incidents and using coercive measures, which reduced injuries to both service users and staff. They also described how they minimized coercive measures through communication.

A lot of the time, it works really well. And it did that one morning for a guy with psychosis. We knew he found it easier to relate to Anna, the social worker. So we asked Anna, “Can you sit with this guy for a while?”. “Sure”; she felt that she knew this guy pretty well too. She sat down calmly and found out what made him so upset and then it was OK. (Manager 3)

According to some ward managers and staff, some staff members had a disinterest or took a punitive approach towards service users. Sometimes, staff members intervened when other staff members were being too harsh, by saying, “Take it easy, she’s not well”. When a service user only attacked furniture or other objects, and not people, the staff had more time to plan how to intervene in a way that would minimize injuries to the service user or themselves, and without offending the service user more than necessary. If mechanical restrains had to be used, it was important to communicate with the service user.

He established the best contact when he stood by her head and then she recognized him as well […] She begged and pleaded with him all the time that he would help her, because she felt that she was going to be killed by everyone. She, yes… it was like she had someone chasing her that would kill her. So it helped that she recognized him […] that staff member. (Staff 2)

It was also regarded as important to speak with service users after coercive measures had been taken to reduce the risk of the event causing psychological problems for the service user or damaging the relationship between the service user and staff.

Many service users reported having negative experiences of coercive measures and experiences of these being used punitively, and that women received sexist comments. Patients also reported instances of aggressive staff who had frightened service users. Most of the above incidents occurred for one or two decades ago. One service user was more afraid of being in hospital in the 90s than today. Even today, however, some service users were afraid of coercive measures and to ask about the most recent measures used. Despite witnessing some coercive measures, other service users were more concerned about violence in society because they perceived the ward as a safer place than society.

I have been in some places where they had to wrestle people down, but no… Out in society I have seen a lot of violence, but not in the wards. But sometimes they have to wrestle people down when they’re deep in their psychosis; they have to give them an injection in the buttocks. (Service user 3)

## Discussion

Our results indicate that an inclusive ward milieu from the stakeholders’ perspective enables service users to be engaged in their own care and encourages sensible communication between service users and staff. In this inclusive environment, the ward functions as a meeting place, the health care hierarchy is toned down, and staff approach service users in a more equal way. Services need to meet the needs of service users, and allow them to remain in care and be free from coercion, violence, and injuries. Stakeholders highlighted several positive features of today’s inpatient psychiatric care, especially concerning the development seen in recent decades; at the same time, they described a reality that is relatively far from the ideals they expressed. There were also differences in the reported experiences of service users compared to staff and ward managers. Only service users spoke about the patient community as a resource. Staff and ward managers did not see this, they emphasized the staffing level and staff competence, which were not as important for the service users. Service users described the ward as a good asylum, despite criticizing the treatment, access to care, and opportunity to participate in care. All stakeholders were aware of the problem of existing negative views of psychiatric patients, but staff did not talk about the large differences in living conditions between staff and service users, differences that service users saw as problematic since they could not achieve a mainstream lifestyle. Service users were impressed by the ability of certain staff members to communicate with desperate or angry service users. All three categories of stakeholders also reported that some staff members used the “rigid old-fashioned frameworks” to approach caregiving, that they could behave harshly towards service users, and risked provoking service users to violence, leading to coercive measures.

Staff and managers reported difficulties motivating service users to participate in care. Service users wanted to participate, but they did not always understand the content of the decided care. They reported disruptive care without any care plan, and not having access to care when they needed it. In a study conducted in the context of forensic psychiatric care, service users described the concept of patient participation as having three categories: influence, confidence, and own responsibility [40]. Influence was related to good communication and involvement in care, which is very close to the sub-theme of sensible communication in the present study, except that the service users did not feel that they were involved. In our study, service users wanted to take more responsibility, but this was difficult and they did not have confidence in the care process because they seldom had a care plan. Mutual care planning can give hope and offer feelings of safety [34], but service users described experiencing disruptive care. Better care planning would also strengthen the work in prevention of violence since factors outside the ward also can be a trigger for conflicts, violence, and coercive measures [25]. In a previous study, we found that staff seldom reported factors outside the hospital as triggers of violence, and mainly reported patients’ characteristics, staff giving a negative message or decision, staff refusing a service user’s request, and care situations [41]. In the present study, staff and managers rarely talked about the whole care process or the service users’ situation in relation to other services or their social situation outside the hospital.

Staff and ward managers were aware of the importance of building good relationships with service users, as they believed that reciprocal relationships can prevent violence. They seemed to express less paternalistic and more reciprocal views in this study than in a previous study from the same area of Sweden [42]. However, staff and ward managers had a more positive view about their own interaction with service users than did the service users. According to service users, staff rarely took the initiative to start conversations or implement activities. Service users saw co-service users as a possible resource, and realized that they themselves could help others. Staff only highlighted problems with the patient community, and did not consider the possibility that the ward could be a social community that could support recovery and safety [13]. All stakeholders reported in our previous studies that coercive measures were avoided by using alternative interventions, but few managers or staff described a conscious use of evidence-based programs [33, 34, 41]. Around half of the hospitals in Finland have been reported to use interventions from both Safewards and Six Core Strategies [43]. Based on our experience, the use of these intervention programs is significantly lower in Sweden, but there are no data to support this view. In the present and our other studies, staff did not give any references to, or clear descriptions of, evidence-based interventions; however, we interpreted some descriptions as a use of calming-down and de-escalation techniques [33, 41]. Implementing these techniques taken from Safewards could strengthen the secondary prevention of violence and recovery in these settings [25].

Service users reported many problems concerning social justice. They described disruptive care and not having access to care when they needed it. Not having access to care is a problem internationally, and leads to higher rates of mortality and reduced life expectancy [44]. Managers and staff described how they tried to minimize physical and mental injuries to service users during coercive measures, which can be regarded as a good tertiary prevention measure. Service users have described incidents of violence and coercive measures in a way that can be interpreted as traumatizing experiences. At the same time, many service users were more concerned about the violence in society than within the institution, and perceived the ward as a safe place [34]. The issue of minimizing coercion has also been highlighted by policymakers in Europe [4], and psychiatric services are expected to make efforts to reduce coercion in accordance with the Convention on the Rights of Persons with Disabilities [27]. However, this declaration has not significantly affected everyday psychiatric inpatient care in Sweden or in other locations [45]. A good start could be to implement evidence-based programs in co-creation, and draw upon the experiences of both service users [28, 46] and staff members [47]. Both staff and ward managers expressed the desire to have more support from the management; thus trust in the management’s understanding of the problem of violence and their ability to deal with it seemed to be low, which was also found in the other studies of this research project [34, 41, 48]. This disappointment may explain why nearly one-fifth of staff reported value incongruence, not sharing the organization’s values [48]. If psychiatric services want service users to feel socially included and safe, the management needs to act as a role model for social inclusion, for both staff and service users. Using evidence-based programs, such as the Six Core Strategies, management could help to form a robust organization, take back the initiative, and create trust.

### Strengths and limitations

One strength of this study is that we mapped social inclusion from the perspective of service users, staff, and ward managers. This enabled us to use the framework method and better understand different stakeholders’ views on social inclusion in inpatient care. The previously published articles, done with inductive analysis, provide a more detailed description of staff’s and managers’ own views on safety and ethics [33] and patients’ own views on safety and unsafety [34]. The present study has a more theoretical approach focusing on dimensions of social inclusion found in the interviews, and therefore provides a different perspective on the situation in psychiatric wards than the previously published articles. The quotations we use are not published in the previous articles, which means that these three publications complement each other. Our interview guides were designed for the previous two studies [33, 34] and were concerned with interactions in psychiatric inpatient care, including aggressive interactions, but did not focus on social inclusion specifically. It could be considered a strength that those who were interviewed did not know that we were researching the topic of social inclusion, and thus their responses were not influenced by this awareness. On the other hand, the main limitation of this study is that the interview guides were not designed according to the aim of the present study and thus we did not optimally cover the entire area of social inclusion. For example, the interview guides did not include specific questions about the care process; therefore, we collected less information on participation in relation to the other values. Since this is a deductive analysis, the analysis has only included data that we believe have a relationship to our dimensions in social inclusion where the views of different stakeholders are contrasted.

## Conclusions

A contribution of this study is that we from the model of social inclusion by Clifton et al. [7] developed a tentative model of social inclusion integrated with violence prevention, based on the values of participation, reciprocity, and social justice. Using our model gave us new insights not gained in our previous inductive analyses of the same interview data. By applying the model on empirical data we identified several factors that can support or disrupt the process to create a safe ward where service users can feel socially included.

In delivering inpatient psychiatric care and striving to prevent violent incidents, it could be helpful to be aware that staff and service users may have different views on the reciprocity of their mutual relationships, and that users may experience a lack of social justice. It is important to plan what will happen after discharge together with the user, who, due to harsh living conditions, may be more concerned about the risk of violence in the community than the violence experienced as inpatients. There seems to be a need for greater focus on service users’ situation in relation to other services and their social situation outside the hospital. Staff and ward managers need support from the management to foster a sense of community in the ward, for example by providing support to use evidence-based prevention programs. This was the first study to use a model to assess social inclusion and empirically study psychiatric inpatient care, and there is a need for more empirical and theoretical studies in this area [49].

## Supplementary Information

**Additional file 1.** Interview guides for interviews with ward managers and focus group interviews with staff.

## Data Availability

Data are not available since this could compromise the individual privacy of participants. The data are stored at the University Health Care Research Center, Region Örebro County, and may be requested by other researchers.

## Abbreviations

WHO: World Health Organization
PICU: Psychiatric Intensive Care Unit
