# Supplementary material for: Social inclusion and violence prevention in psychiatric inpatient care. A qualitative interview study with service users, staff members and ward managers
Source: BMC Health Serv Res. 2021 Nov 20;21:1255. doi: 10.1186/s12913-021-07178-6 (PMC8605501; doi:10.1186/s12913-021-07178-6)
Supplement: Supplementary file 1 — Additional file 1. Interview guides for interviews with ward managers and focus group interviews with staff. [file 12913_2021_7178_MOESM1_ESM.docx]

# Interview guides

Ward managers, p 1.

Focus group with staff, p 3.

## Ward managers

### Background questions

**Ethical issues in general**

What forums are there to address ethical issues?

What training have you and the staff received in terms of ethics?

How much supervision and guidance do staff have in matters of ethics?

Are there policy documents, rules and routines regarding ethics?

Have you and the staff been involved in producing, or influencing the content, of these documents?

In what way are these used in daily work?

How do you get patients to be involved in participating in their care?

**Security/safety issues in general**

What forums are there to address issues of security/safety and coercion against patients?

What training have you and the staff received in terms of security/safety issues?

How much supervision and guidance do staff have in matters of security/safety issues?

Are there policy documents, rules and routines regarding security/safety issues.

Have you and the staff been involved in producing, or influencing the content, of these documents?

In what way are these used in daily work?

### Core questions

**Practical handling of violent situations**

Are there guidelines for how to interact with patients in aggressive and violent situations?

How do you handle meetings where you see a risk of aggression from patients?

Do staff manage to remain calm in situations of aggression and violence? Give examples of successful and unsuccessful situations.

What should staff do, and not do, in aggressive or violent situations?

How are you dealing with violent incidents afterwards with staff who have participated, a) in the working group, b) with management, c) with patients?

How are staff affected by aggressive and violent incidents (health, work ethics, efficiency and quality of care)?

Are there staff who are more fit to handling difficult situations and who therefore become the ones who mainly take care of situations with aggression and violence? (Including the gender perspective)

**Critical incidents**

*If there is time, we ask about a maximum of two incidents that the respondent perceived as most violent or threatening during the last two years and what happened then.*

A: Give a brief description of the violent or threatening event (including when and where)

B: Possible follow-up questions:

1. What happened immediately before the incident?
2. Are there factors that go back in time that could explain the incident?
3. What happened during the incident?
4. What happened immediately after the incident?
5. What consequences did the incident have for you (and others)?
6. What steps did you (and others) take to deal with the problems caused by the incident?
7. Do you have suggestions on what could be changed to avoid similar incidents in the future?

## Focus groups with staff

### Background questions

**Ethical issues in general**

What forums are there to address ethical issues?

What training have you received in terms of ethics?

How much supervision and guidance do you receive in ethical issues?

Are there policy documents, rules and routines regarding ethics that you benefit from in your daily work?

Have you been involved in producing, or influencing the content, of these documents?

In what way are these used in daily work?

How do you make patients get involved in their care?

**Security/safety issues in general**

What forums are there to address issues of security/safety and coercion against patients?

What training have you received in terms of security/safety?

How much supervision and guidance do you receive in matters of security/safety?

Are there policy documents, rules and routines regarding security/safety that you benefit from in your daily work?

Have you been involved in producing, or influencing the content, of these documents?

In what way are these used in daily work?

### Core questions

**Practical handling of violent situations**

*Main Question: What happens when the alarm goes off in the event of an acute violent incident with a patient/s?*

Do staff manage to remain calm in situations of aggression and violence? Give examples of successful and unsuccessful situations.

To deny patients something they want (or handle other conflicts); how do you do it?

How do you handle meetings where you see a risk of aggression from patients?

Are there guidelines for how to address aggression or violence from patients?

What to do, what should you not do, in aggressive and violent situations?

How are you dealing with violent incidents afterwards with staff who have participated, a) in the working group, with b) management and c) patients?

What role does the ward manager have in critical incidents?

How are you as a staff affected by aggressive and violent situations (health, work ethics, efficiency and quality of care)?

Are there staff who are more fit to handling difficult situations and who therefore become the ones who mainly take care of situations with aggression and violence? (Including the gender perspective)
